# Bullying in the Russian Secondary School: Predictive Analysis of Victimization

**DOI:** 10.3389/fpsyg.2021.644653

**Published:** 2021-07-29

**Authors:** Garen Avanesian, Liudmila Dikaya, Alexander Bermous, Sergey Kochkin, Vladimir Kirik, Valeria Egorova, Irina Abkadyrova

**Affiliations:** ^1^Academy of Psychology and Educational Sciences, Southern Federal University, Rostov-on-Don, Russia; ^2^Department of Higher Mathematics, Northern (Arctic) Federal University, Arkhangelsk, Russia; ^3^International Institute of Interdisciplinary Education and Ibero-American Studies, Southern Federal University, Rostov-on-Don, Russia

**Keywords:** bullying victims, adolescents, Russian secondary school, statistical analysis, learning outcomes, psychological environment, emotional states, psychosocial traits

## Abstract

**Background:** Bullying has been recognized as an important risk factor for personal development in adolescence. Although numerous studies report high prevalence of bullying in Russian schools, limited research was based on the large-scale, nationally representative analysis, which highlights the lack of findings applicable to the national context.

**Objective:** This study aims to address the following research questions: (1) What is the bullying victimization prevalence in Russian secondary schools? (2) What is the socio-demographic profile of the bullying victims? (3) To what extent do learning outcomes in core subject domains predict bullying? (4) How does psychological climate at school affect the occurrence of bullying? (5) Which emotional states do bullying victims typically display? (6) Which psychosocial traits are the most common for bullying victims?

**Data and Methods:** The study adopts the statistical analysis of the Programme for International Student Assessment (PISA) data in Russia. The final sample consists of 6,249 children aged 15 years who answered the bullying questions. K-means clustering approach was adopted to identify schoolchildren who should be classified as bullying victims amongst those who have reported bullying. Logistic regression was used to estimate the probability change of bullying under different psychosocial factors and examine the effect of bullying on the emotional states of the victims.

**Results:** The results of the study reveal that 16% of children are victims of bullying in the Russian secondary school. Bullying is strongly associated with learning outcomes in reading, thus outlining that low performers are at risk of severe victimization. Bullying is also contingent on the psychological climate and tends to develop more frequently in a competitive environment. The findings outline that bullying increases negative feelings such as misery, sadness, and life dissatisfaction amongst its victims, making a substantial footprint on their lives. Logically, bullying victims are less likely to feel happy and joyful. Finally, it was revealed that bullying victims do not tend to share negative attitudes to the *per se*, which identifies directions for future research in this domain.

**Implications:** Instead of dealing with the consequences of bullying, prevention strategies should aim at facilitating a positive environment at school, thus addressing the problem.

## Introduction

In 2015, while approving Sustainable Development Goals (SDG, the United Nations General Assembly prioritized inclusive, equitable, and quality education for all. Later in the same year, Incheon declaration committed to “addressing all forms of exclusion and marginalization, disparities and inequalities in access, and participation and learning outcomes,” which goes in line with SDG target 4.a, to build education environments that are “child, disability, and gender sensitive and provide safe, non-violent, inclusive, and effective learning environments for all” (UN, 2015). Therefore, inclusiveness in education refers to the fundamental human rights of every schoolchild, and the efforts of education stakeholders should strive to satisfy the needs for a safe and psychologically comfortable learning environment. On the other hand, the psychological needs for inclusive and safe education are often not met in many contexts where children still become victims of bullying, abuse, or even violence.

Bullying is common amongst teenagers, as this group frequently demonstrates contradictive aspirations to be independent on the one hand and gain social acknowledgment and prestige on the other (Adler and Adler, 1995; LaFontana and Cillessen, 2002; Lease et al., 2002; Dijkstra et al., 2008). Furthermore, research confirms that often times, bullying takes place amongst classmates (Salmivalli and Voeten, 2004; Pečjak and Pirc, 2017; Nesterova and Grishina, 2018). It occurs as a result of asymmetric power balance between the perpetrators and victims. Bullying is characterized by conscious and rational humiliation, aggression, or even violence toward others, which inevitably leads to a decreased self-esteem and victimization of those at whom it is directed (Krivtsova et al., 2016; Grishina, 2017). Accounting for a variety of definitions, we look at bullying as a “longstanding violence, physical or psychological, conducted by an individual or a group, and directed against an individual who is not able to defend himself in the actual situation” (Roland, 1993, p. 16).

The research focuses on bullying victims with the aim to draw their psychosocial portrait and predict the factors behind bullying. Although the phenomenon of bullying is widely covered in the international body of work, scarce scientific evidence on the issue was produced in the Russian academic literature. Existing studies suggest that the prevalence of bullying in Russian schools is high, and on an average every one out of four schoolchildren faces risks of becoming a bullying victim (Gorlova and Kuznetsova, 2019; Rean and Novikova, 2019; Shalaginova et al., 2019). However, there is a lack of research on bullying in Russian schools that employ representative data analysis, which highlights a sizeable gap in the national body of work and emphasizes the need for data-driven research in this domain.

To analyze victimization caused by bullying, we use the data collected from the latest 2018 round of the Programme for International Student Assessment (PISA), which measures the learning outcomes of the students in the last year of lower secondary school. This school-based survey program supplies researchers with a vast number of dimensions to test their hypotheses, including questions to monitor bullying frequency in schools amongst adolescents.

### Unique Contributions

The current study explores cognitive, emotional, and psychosocial factors associated with bullying amongst students in the Russian secondary school. Adopting nationally representative data from PISA-2018 in Russia, the findings of the present research contribute to the growing body of knowledge regarding bullying in the Russian secondary school. Being also first-of-its-kind in terms of geographical coverage, the analysis of bullying victimization carried out in this study generates data-driven proposals for efficient bullying prevention programs.

## Literature Review

The relevance of research on bullying is high because this destructive behavior is widely spread amongst children and adolescents all over the world (Zych et al., 2017). Negative consequences of bullying are self-explanatory, causing psychological traumas and stigma amongst the victims; evidence suggests that it can even affect the academic achievements of a child (Schwartz et al., 2005; Nakamoto and Schwartz, 2010). Bullying is well-studied in the international body of work (Fedunina and Sugizaki, 2012; Swearer and Hymel, 2015a; Bethel, 2016; Espelage et al., 2016; Grishina, 2017; Naumova and Efimova, 2018; Peng et al., 2020; Vorontsov, 2020). This subject has caused a growing interest amongst Russian scholars in the recent decade (Nesterova and Grishina, 2018; Shalaginova et al., 2019; Vorontsov, 2020). Moreover, existing evidence suggests that bullying in Russian schools tends to occur more frequently than in economically developed democratic countries (Rean and Novikova, 2019).

As it has been mentioned earlier, there are many different approaches to define and study bullying. However, they can be integrated into three major groups: dispositional, which aims to examine individual characteristics of actors involved in bullying; temporal, which focuses on risks related to the time when people act as a bully and a victim; and contextual, which emphasizes the role of environment in triggering bullying (Bochaver and Khlomov, 2013). When it comes to bullying typology, it is suggested to differentiate bullying between direct, which can take verbal or physical aggression, and indirect, that refers to psychological or relational expression (Espelage and Swearer, 2003; Doll and Swearer, 2006). Considerable growth of internet penetration in contemporary societies contributed to the spread of cyberbullying, which highlights aggressive and offensive behavior on the internet (Schott and Søndergaard, 2014; Ekimova and Zalaldinova, 2015). Bullying always involves at least three types of social actors: a bully, a victim, and a bystander. A bully is defined as a person who perpetrates psychological pressure or physical power over the victim (Rose et al., 2011). A person incapable of self-defense appears to be a victim. Finally, bystanders are defined as individuals who either reinforce a bully or defend a victim (Marini et al., 2001; Salmivalli et al., 2011; Butenko and Sidorenko, 2015; Nesterova and Grishina, 2018).

The factors behind bullying refer to various aspects of the social and psychological environment. In this context, the appearance of a person can exert a profound influence on bullying. Frequently, children suffering from overweight or those physically less developed, children unhappy with the way they look tend to be bullying victims (Janssen et al., 2004; Griffiths et al., 2006; Faris and Felmlee, 2014). Gender leaves a specific footprint on bullying, too. The analysis of the existing body of academic literature highlights the gender differences favoring boys in direct physical aggression and trivial gender differences in the relational aggression (Card et al., 2008; Stubbs-Richardson et al., 2018). Several studies suggest that bullying is inversely associated with socioeconomic status, meaning that children from low-status groups have a higher exposure to becoming a bully or a victim of bullying (Tippett and Wolke, 2015; Nesterova and Grishina, 2018; Ryumina, 2018).

However, socio-demographic or economic factors cannot solely explain the occurrence of bullying. Psychosocial features at the individual or group level refer to another critical group of factors behind bullying. When it comes to the school environment, bullying is highly likely to be triggered by low empathy and tolerance levels observed in some children, as well as by high levels of aggression. Some students in the conflict tend to adopt competitive strategies, thus prioritizing the satisfaction of personal needs at the expense of others (Huseynova and Enikolopov, 2014; Shalaginova et al., 2019). Studies indicate that disciplinary climate and the feeling of belonging amongst children are of particular importance because bullying is less frequent in schools where disciplinary aspects (attendance, attention, and involvement) are positive, and children feel connected with others (Nesterova and Grishina, 2018; Novikova and Rean, 2019). Sometimes bullying can be exacerbated by ignorance on the part of school management, which reacts to physical violence only, thus underestimating the importance of such secondary indicators as rumors or verbal feud (Olweus, 1997; Lane, 2001; Petrosyants, 2011).

Family environment also affects the propensity of a child to become a victim of bullying at school. In this regard, the aggression of parents can prompt the role of a bully in a child, and aggression from siblings within the family could further victimize a child at school (Volikova and Kalinkina, 2015; Nesterova and Grishina, 2018). Sometimes, in contrast, a child being a victim of bullying at school expresses personal aggression toward younger siblings and thus becomes a bully in other settings, which was defined in the academic literature as a bully–victim (Salmivalli, 2013; Swearer and Hymel, 2015b).

Furthermore, mounting evidence suggests that bullying can severely impact the psychological well-being and emotional states of the victims. Several studies prove that victims of bullying tend to have a lower self-esteem and decreased life satisfaction. Socially, they tend to be very unconfident, exhibiting a higher fear of failure and leaving their social ambitions and claims unpronounced (Haynie et al., 2001; Lane, 2001; Salmivalli and Nieminen, 2002; Striegel-Moore et al., 2002; Glazman, 2009; Kochel et al., 2012; Rodkin et al., 2015). They also report higher anxiety, solitude, suicidal thoughts, the feeling of being socially excluded, and other harmful psychosocial conditions (UNESCO, 2018).

Given the harmful effect of bullying on the lives of schoolchildren, relevant stakeholders need to elaborate prevention strategies to provide an inclusive, safe, and psychologically comfortable environment for learners. However, in the Russian context, most measures have been directed at eliminating negative consequences of bullying, reducing the level of aggression, or providing support to victims. On the contrary, a framework based on positive psychology suggests that measures directed at creating a positive psychosocial environment at schools can be more efficient in eliminating bullying as they tackle the cause of the problem instead of dealing with its consequences (Rean and Stavtsev, 2020). If prevention strategies aim to increase self-esteem and motivation of schoolchildren, as well as harmonize social interaction between children and teachers, these strategies have the potential to create a solid basis for positive outcomes that go beyond eliminating bullying.

This study takes a closer look at bullying in the Russian secondary school. Accounting for that, the objective of this work is to identify complex factors that influence the propensity of a schoolchild to become a bullying victim. In addition to that, we aim to take a closer look at the bullying victims to better understand their psychosocial profile.

## Research Gap and Research Questions

### Research Gap

Review of the academic literature contributes to formulating the directions for current analysis and identifying a research gap in the existing body of academic work on bullying. The analysis carried out in this study attempts a novel approach to understand bullying in Russian schools through a scope of complex factors that can condition bullying as well as give an insight into the psychosocial and emotional states of its victims. Most of the research in the Russian context was based on insufficient sampling, which precluded from generalizing results with a national scope. To overcome this limitation, we used the data rendered in the last wave of PISA, which gave a nationally representative sample of more than 6,000 students all over the country.

Furthermore, many studies indicate that bullying affects the academic performance of the victims. However, this question has not yet been conversely addressed. At this point, not much is known about how academic achievement, learning outcomes, or cognitive skills affect the propensity of a child to become a victim of bullying.

### Research Questions

Accounting for the research gap highlighted and in alignment with the study objectives, this analysis attempts to answer the following questions:

- What is the bullying victimization prevalence in Russian secondary schools?
- What is the socio-demographic profile of the bullying victims?
- To what extent do learning outcomes in core subject domains predict bullying?
- How does the psychological climate at school affect the occurrence of bullying?
- Which emotional states do bullying victims typically display?
- Which psychosocial traits are the most common for bullying victims?

## Data and Methods

### Bullying Scale

The bullying scale was introduced to PISA in 2015. The index of exposure to bullying is measured based on the six main items. Data collection is based on the self-assessment of a schoolchild, when respondents need to indicate the frequency with which they experience bullying. Possible answers include “never or almost never,” “a few times a year,” “a few times a month,” and “once a week or more.” The options outlined have corresponding numeric values ranging from 1 to 4, where the highest value indicates the highest frequency. Taken together, the items result in the standardized index with 0 as mean value and 1 as standard deviation across the member countries of the Organization for Economic Co-operation and Development (OECD). Proceeding from this, “positive values on the index indicate students who reported to be more frequently bullied than the average student in OECD countries, while negative values indicate students who reported less frequent exposure to bullying than the average student in OECD countries” (OECD, 2017, p. 135). The OECD reports that the proposed scale was tested in all countries where monitoring is conducted, which resulted in a Cronbach α of 0.88 for OECD countries, 0.83 for all countries, and 0.81 for Russia (OECD, 2017). However, since the analysis does not aim to make comparative inferences about bullying in a crosscultural perspective, it explicitly focuses on the Russian context, creating requirements to reconsider both scale items and the scoring algorithm. To do that, we need to start by exploring how reliable and valid the scale is in relation to the Russian context and then find better ways of aggregating the final index. Moreover, the OECD average cannot be used as a reference for the Russian context, since everyday life, living standards, and school environment of developed high-income countries vary from those of Russia.

With this regard, the second relevant issue refers to the aggregation of the index. As outlined by the PISA 2015 report (OECD, 2017, p. 135), such answer options as “a few times a month” and “once a week or more” were grouped for better “international invariance of the scale.” However, as the international comparison does not form the current research agenda, we decided to avoid merging these options and thus left the scale in the range of 1–4.

Analogous to the index of exposure to bullying suggested by the OECD, we employed standardization procedures to compute the index, where 0 indicates the average exposure of a schoolchild in Russia to bullying, and the range of values potentially varies within plus/minus three standard deviations.

### Statistical Modeling of Data

Statistical analysis was carried out in three main stages. First, we wanted to understand the prevalence of bullying in Russian secondary schools. In other words, the purpose was to estimate the probability of a child becoming a victim of bullying at school. We did not want to produce arbitrary decisions upon selecting a random threshold to distinguish victims of bullying from other students that might experience it occasionally. With this in mind, we conducted cluster analysis that helped identify victims of bullying in the overall number of Russian schoolchildren. This resulted in a binary variable “Victim,” which assigned 1 to students who are victims of bullying and 0 to those who are not.

Inferential analysis went in two main directions. The first one used bullying as a dependent variable and aimed to model factors that could predict it. On the other hand, it was also necessary to understand the scope of reactions that bullying causes in its victims. Therefore, in the second stage of the analysis, bullying served as a predictor, whereas different emotional states or psychosocial traits were considered response variables. This step allowed for a better understanding of the profile and typical characteristics of bullying victims. We chose logistic regression to statistically model these relationships, which allowed for fixing the effects of the predictors on the probabilistic scale. The calculated model has the equation below:

$$\begin{array}{l} log\frac{P\left(Y\right)}{1-P\left(Y\right)}=\alpha +\beta_{1\;}X_{1}+\beta_{2}X_{2}+\ldots +\beta_{n}X_{n}+\;\varepsilon , \end{array}$$

where $log\frac{P\left(Y\right)}{1-P\left(Y\right)}$ is a logarithm of odds ratio that a child is bullied, α is a model constant, *X*_*n*_ is a predictor, β_*n*_ is a coefficient of change associated with it, and ε is an error-term of the model.

The first model aimed to estimate how the learning outcomes of students shape bullying. The second model assessed the effects of the psychological environment in schools. Then we used bullying as a predictor to assess its impact on the emotional states of victims. PISA collects data on the eight emotional states, namely: happiness, joy, cheerfulness, liveliness, pride, misery, sadness, and fearfulness of schoolchildren. Schoolchildren were asked how frequently they experience a specific emotional state with four answer options, such as never, rarely, sometimes, and often. Those who answered “often” while reporting a specific emotional state were coded as 1 in opposition to other schoolchildren coded as 0. The same approach was used to estimate the propensity to different psychosocial traits. However, the responses there were fixed on the 1–4 scale, and depending on the variable, top 20% or bottom 20% were taken as the groups for calculating the effects of bullying on them. These parts of the analysis present a series of models that consisted of only two variables, bullying as a predictor and emotional state or psychosocial trait as an outcome variable.

For all logistic regression models, both dependent and independent variables were transformed into categorical ones, and the results were presented not as regression coefficients but as marginal effects. As logits or odds ratio scales are not informative in summarizing how changes in response variables are associated with changes in predictors, presenting results as differences in probabilities was more meaningful for interpretation. Marginal effects are non-linear and present the magnitude of change on the probability scale. Therefore, depending on the value of predictors, the effect is always bound between 0 and 1. Marginal effects are easy to calculate using the equation below:

$$\begin{array}{l} \;\Delta P=P_{2}\left(Y\right)-P_{1}\left(Y\right),\;P\left(Y\right)=\frac{e^{\sum_{i=0}^{n}\beta_{i}X_{i}}}{1+e^{\sum_{i=0}^{n}\beta_{i}X_{i}}}\\ \Delta P_{X_{i}}=P_{X_{i}=1}\left(Y\right)-P_{X_{i}=0}\left(Y\right),\\ P_{X_{i}}\left(Y\right)=\frac{e^{\alpha +\beta_{1}{\bar{X}}_{1}+{\ldots +\beta }_{i}X_{i}+\ldots +\beta_{n}{\bar{X}}_{n}}}{1+e^{\alpha +\beta_{1}{\bar{X}}_{1}+{\ldots +\beta }_{i}X_{i}+\ldots +\beta_{n}{\bar{X}}_{n}}} \end{array}$$

Data analysis was carried out in *R*, lingua franca of statistical computing.

This project was registered in Open Science Framework (see link here: https://osf.io/vhjr3/).

## Results

### Prevalence and Profile of Victims

In the 2018 PISA wave, 6,249 schoolchildren aged 15 years old in Russia responded to questions related to bullying in a student questionnaire. OECD conceptualized bullying within three core subdimensions: relational, physical, and verbal represented by the scale items (OECD, 2017, p. 135). The analysis suggests that verbal bullying has the highest prevalence in Russian secondary schools. As such, 16% of schoolchildren confessed that other students made fun of them either a few times a month or once a week or more. It is followed by relational bullying expressed in spreading nasty rumors, which was frequently reported by 14% of schoolchildren. Physical bullying expressed by threatening, destroying personal belongings, or pushing and hitting occurs relatively rare, being reported by 3.5% of schoolchildren on average. Disaggregated by sex, the data suggest that across all types of bullying, boys tend to report the occurrence of bullying “once a week or more” more often than girls. The data on bullying prevalence by type, also broken down by sex, are summarized in Table 1.

**Table 1:** Prevalence (%) of bullying types, broken down by sex.

	**Item**	**Never or almost never**			**A few times a year**			**A few times a month**			**Once a week or more**		
		**Boys**	**Girls**	**Total**	**Boys**	**Girls**	**Total**	**Boys**	**Girls**	**Total**	**Boys**	**Girls**	**Total**
1	Other students left me out of things on purpose	49.0	53.0	51.1	25.5	25.6	25.6	14.6	13.0	13.8	10.9	8.3	9.6
2	Other students made fun of me	56.5	64.4	60.6	25.3	21.8	23.5	11.3	9.4	10.3	6.9	4.4	5.6
3	I was threatened by other students	71.2	82.8	77.2	14.6	9.5	12.0	9.8	5.9	7.8	4.4	1.8	3.1
4	Other students took away or destroyed things that belong to me	70.9	78.7	74.9	14.0	12.7	13.4	9.7	5.9	7.8	5.4	2.7	4.0
5	I got hit or pushed around by other students	75.2	84.5	80.0	10.9	8.1	9.4	9.2	5.6	7.3	4.7	1.8	3.2
6	Other students spread nasty rumors about me	67.6	71.4	65.9	16.1	17.5	16.8	9.6	8.1	8.8	6.7	3.0	4.8

*Source: Calculations of the authors based on PISA-2018 in Russia*.

Reliability analysis of the bullying scale based on the Russian data has revealed that the standardized Cronbach αof the six-item scale accounts for 0.88. Although the scale demonstrates high overall reliability, it is seen from Table 2 that dropping the first item, “Other students left me out of things on purpose,” would improve reliability by increasing the value of the standardized Cronbach α from 0.88 to 0.91.

**Table 2:** Reliability and validity analysis of the scale of the exposure to bullying on the Russian PISA data.

**No**	**Item**	**Type of bullying**	**Reliability if an item is dropped**	**Correlation with the first principal component**
1	Other students left me out of things on purpose	Relational	0.91	0.28
2	Other students made fun of me	Verbal	0.87	0.61
3	I was threatened by other students	Verbal/Physical	0.85	0.76
4	Other students took away or destroyed things that belong to me	Physical	0.85	0.73
5	I got hit or pushed around by other students	Physical	0.85	0.74
6	Other students spread nasty rumors about me	Relational	0.86	0.70
	Overall Cronbach Alpha		0.88	

*Source: Calculations of the authors based on PISA-2018 in Russia*.

In order to understand the validity of the scale, we performed principal component analysis (PCA). The PCA results confirm that the first component explains 64% of the total variance, which means that there is no need to divide the composite index into subdimensions following the bullying types. In other words, the items load well on the unidimensional concept with the eigenvalue equal to 3.8. However, while items two to six obtained Pearson correlation coefficients with the principal component above 0.6, the first item scored just below 0.3. Consequently, the reliability and validity of the bullying scale in the Russian language provide sufficient statistical reason to exclude the first item from the analysis. Taking the arithmetic mean of five items in this case would result in the higher weight of items related to the physical bullying in the final score. Nonetheless, as the remaining items establish a high correlation with the first principal component, as shown on Table 2, it gives a solid statistical ground to aggregate a final score in a one-dimensional concept instead of aggregating by conceptual subdimensions (that correspond to different bullying types) and then taking their mean value. These statistical results might also have a cultural reasoning behind: in the Russian context, physical bullying indeed has a higher relevance in comparison to other types, which explains the low reliability and validity scores for the first item, which represents relational bullying. As such, some studies emphasize a particular importance of physical bullying in the Russian context, articulating that in opposition to more subjective by their nature relational and verbal forms of bullying that indeed occur more frequently, physical aggression is more explicit (Khanolainen et al., 2020). Therefore, the suggested way of aggregating the scale could help to estimate the prevalence of severe victimization. In this context, the precise estimation indeed should go beyond reporting the prevalence of different bullying types measured by the scale items. For understanding overall prevalence, one needs to approach the topic from the perspective of the aggregated score. As bullying is a relative scale that fixes personal attitudes, perceptions, and reflections, it makes sense to standardize the indicator to position students relative to each other. The mean value thus was transformed to 0, whereas 1 indicated a standard deviation across the Russian sample. With regard to this, index values above 0 indicated that all school children who are bullied more than a schoolchild in Russia are bullied on average.

On the other hand, negative values allowed for identifying schoolchildren who experience bullying more rarely than a schoolchild on average. The association of the index calculated for the Russian sample with the original bullying index of OECD showed a statistically significant correlation at the level of 0.81. However, this high value should not be misinterpreted as it primarily means that 34% of the variance remains unexplained in this bivariate association (as squared Pearson R gives us a coefficient of determination). This confirms that our choice of producing a separate index for the Russian data was justified.

**Figure 1:**
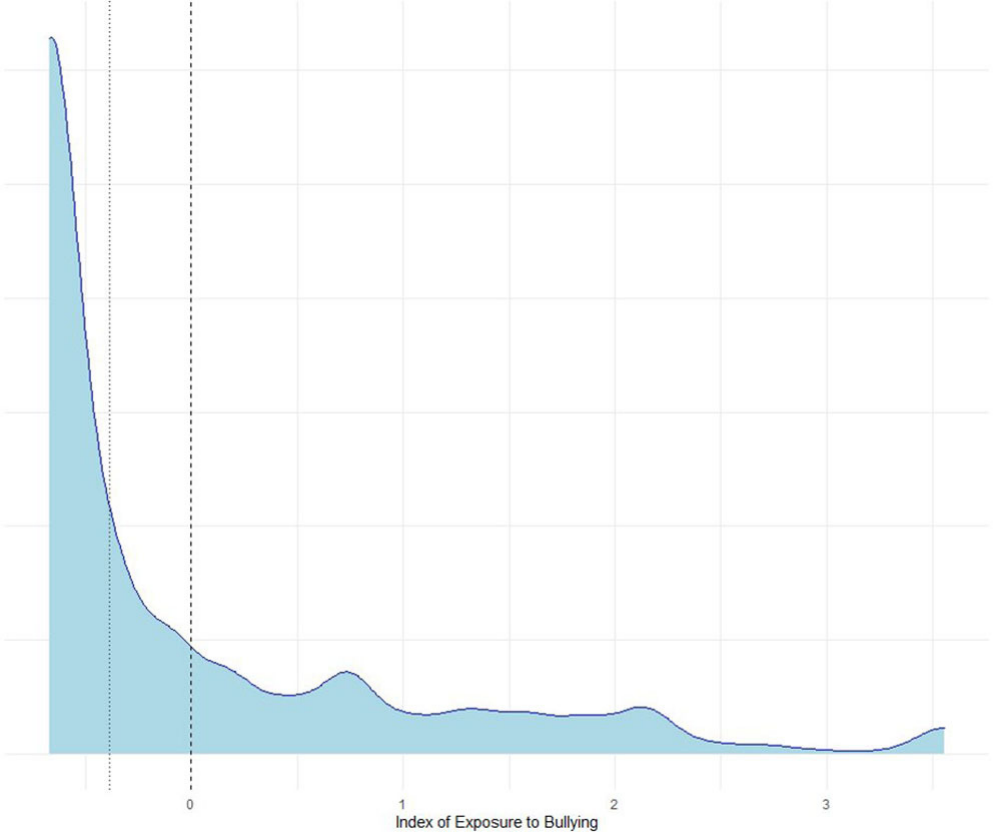
Probability density plot of the index of exposure to bullying. Note: dashed line indicated mean value, whereas the dotted one, median Source: Calculations of the authors based on PISA-2018 in Russia.

However, one question remained open: how to identify schoolchildren that are actual victims of bullying. The resulting index varied from −0.67 to 3.56, outlining a high heterogeneity in distributing the scores. As Figure 1 shows, the distribution is positively skewed, with a median value equal to −0.39, which means that at least 50% of all schoolchildren in Russia are bullied less than average. In turn, bullying that reaches the average maximum value of the country can occur with the probability that accounts for ~70%. Therefore, the division of schoolchildren into those for whom bullying is something that happens occasionally and those who are victims of it should inevitably be defined by statistical distribution logic. Figure 1 presents the probability density plot of index of exposure to bullying derived from the five items of the bullying scale. The distribution is both positively skewed and multimodal. Therefore, we suggest that the demarcation between the two groups should somehow account for the peaks. The first peak representing the index values that are approximately equal to 0.6 is of particular interest. However, to avoid arbitrary decisions based on a random assignment of the threshold value, we decided to adopt k-means clustering.

In many ways, the clustering results confirmed our assumptions: the multimodality of distribution explicitly demarcates the borders between two groups. The algorithm classified those who obtained an index score higher than 0.67 as bullying victims. It is worth mentioning that this cohort accounts for 16% of all schoolchildren, which means that every one out of six schoolchildren in Russia is a victim of bullying.

Profiling of the bullied victims forms another critical pillar of the analysis. It is essential to understand the composition of the group that experiences a high risk of exclusion. Though bullied students have a very heterogeneous background, we can still identify a few distinct patterns while looking at cohorts that comprise bullying victims. First, bullying in school occurs more frequently with boys than with girls, every two out of three bullying victims being male schoolchildren. It is also clear that victims of bullying carry psychological stigmatization associated with their status in society. More than 43% of the schoolchildren bullied belong to low-status groups by the PISA index of economic, social, and cultural capital. Finally, in ~70% of all cases, bullying victims reside in villages or towns with a population below 100,000 inhabitants. However, these numbers should not be interpreted in the causal perspective. Profiling helps us draw a portrait of a particular group by key socio-demographic dimensions; however, it presents descriptive statistics that in many ways could be affected by population distribution.

### Bullying and the Associated Phenomena

**Figure 2:**
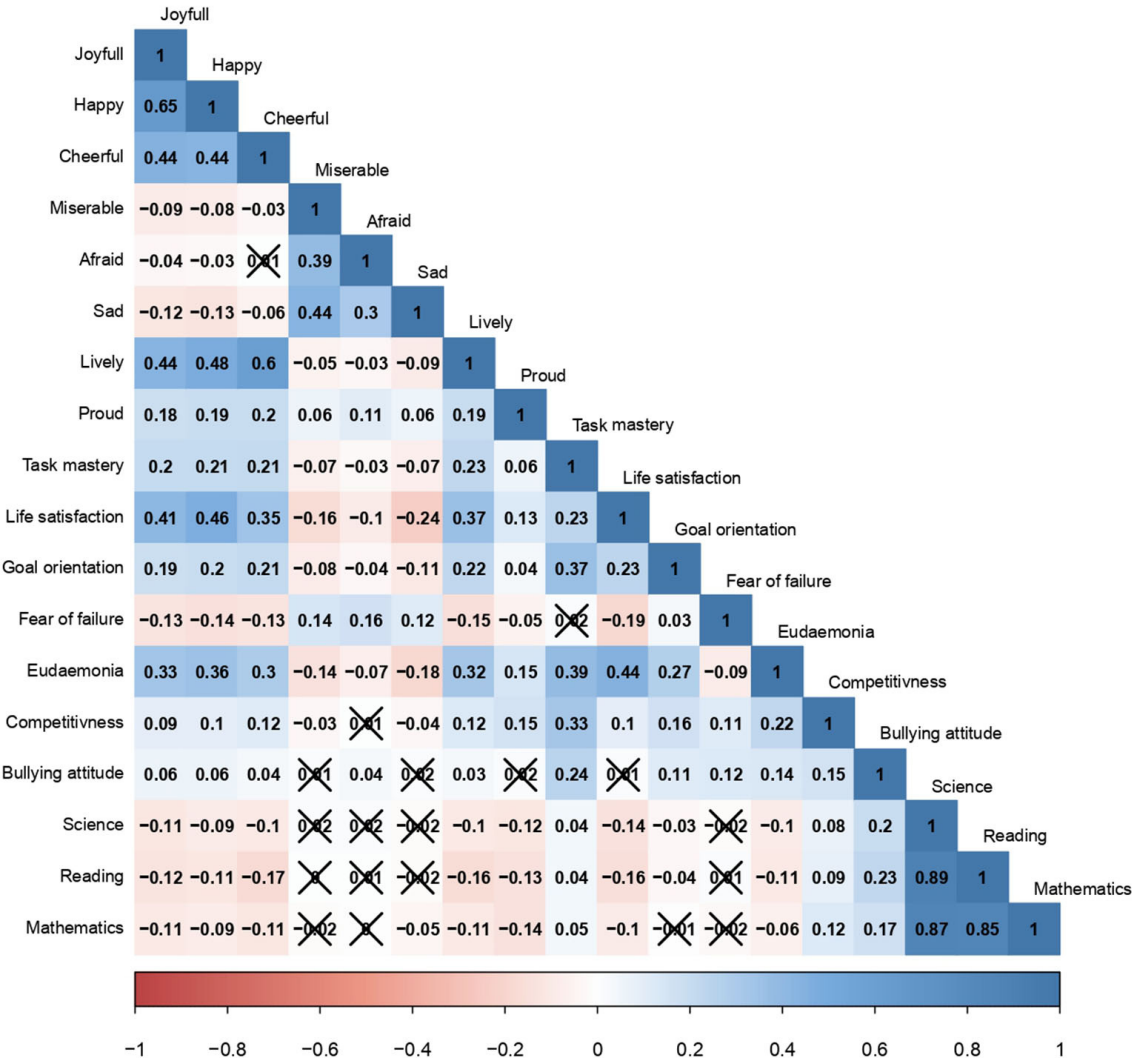
Correlation matrix (Spearman's rank correlation coefficient) of variables associated with bullying. Note: crossed cells refer to correlations that are not statistically significant. Source: Calculations of the authors based on PISA-2018 in Russia.

This study explores the relation of bullying with the number of characteristics that can be grouped into four categories. The first one refers to learning outcomes and is comprised of skills in readings, mathematics, and science. Disciplinary climate at school, perceived cooperativeness and competitiveness of the school environment, and school belonging form the second group of the variables and denote the psychological environment at school. The third pillar of the analysis explores the impact of bullying on the propensity of frequently experiencing emotional states such as happiness, joy, cheerfulness, liveliness, pride, misery, sadness, and fearfulness. Finally, the fourth category is represented by the impact of bullying on diverse psychosocial characteristics and traits, which are life satisfaction, eudaemonia, fear of failure, task mastery, personal competitiveness, goal orientation, and attitude to bullying. Table 3 provides the detailed description of the items of PISA questionnaire that intend to measure the outlined phenomena, as well as reports summary statistics on the Russian sample. For measuring the association of the variables outlined above with bullying, all in all 17 logistic regression models were calculated. The first model explored the effect of learning outcomes on bullying, whereas the second one assessed the impact of the psychological environment in school on the occurrence of bullying. Finally, 15 additional models explored how bullying predicts the probability of experiencing a certain emotional state or psychosocial trait. Correlations matrix in Figure 2 shows the associations between all 25 variables used in the study.

**Table 3:** Summary statistics of the variables.

**No**	**Item**	**Strongly disagree**			**Disagree**			**Agree**			**Strongly agree**		
		**Boys**	**Girls**	**Total**	**Boys**	**Girls**	**Total**	**Boys**	**Girls**	**Total**	**Boys**	**Girls**	**Total**
**Task mastery**													
1	I find satisfaction in working as hard as I can.	8.6	5.5	7.0	28.0	29.8	28.9	52.2	55.8	54.1	11.2	8.9	10.0
2	Once I start a task, I persist until it is finished.	4.0	2.8	3.4	23.7	24.0	23.9	54.6	58.0	56.4	17.6	15.2	16.4
3	Part of the enjoyment I get from doing things is when I improve on my past performance.	4.4	2.8	3.6	15.6	13.0	14.3	63.4	68.6	66.1	16.5	15.7	16.1
4	If I am not good at something, I would rather keep struggling to master it than move on to something I may	5.6	3.9	4.7	20.6	23.0	21.8	56.1	57.7	56.9	17.8	15.4	16.6
**Fear of failure**													
1	When I am failing, I worry about what others think of me.	17.4	11.7	14.5	33.0	32.9	32.9	40.1	44.0	42.1	9.5	11.5	10.5
2	When I am failing, I am afraid that I might not have enough talent.	15.8	9.6	12.6	42.9	35.3	39.0	34.1	44.0	39.2	7.2	11.2	9.2
3	When I am failing, this makes me doubt my plans for the future.	18.6	11.4	14.9	37.4	35.1	36.2	34.9	41.6	38.3	9.2	11.9	10.6
**Eudaemonia**													
1	My life has clear meaning or purpose.	7.4	6.1	67.3	16.9	22.6	19.8	50.8	53.6	52.3	24.8	17.7	21.2
2	I have discovered a satisfactory meaning in life.	5.7	5.9	5.8	23.5	29.7	26.7	50.3	49.7	50.0	20.5	14.6	17.5
3	I have a clear sense of what gives meaning to my life.	6.1	6.1	6.1	19.1	22.7	20.9	52.4	53.5	53.0	22.4	17.7	20.0
**Personal competitiveness**													
1	I enjoy working in situations involving competition with others.	11.0	7.6	9.2	19.2	33.1	26.3	49.6	47.1	48.3	20.2	12.2	16.1
2	It is important for me to perform better than other people on a task.	7.6	5.1	6.3	31.5	33.1	32.3	43.4	45.4	44.4	17.5	16.3	16.9
3	I try harder when I'm in competition with other people.	8.3	5.9	7.1	21.1	29.1	25.2	47.3	48.4	47.9	23.3	16.6	19.9
**No**	**Item**	**Never**			**Rarely**			**Sometimes**			**Often**		
		**Boys**	**Girls**	**Total**	**Boys**	**Girls**	**Total**	**Boys**	**Girls**	**Total**	**Boys**	**Girls**	**Total**
**Emotions**													
1	How often do you feel as described below? Joyful	2.7	1.3	2.0	9.4	8.0	8.7	39.9	42.9	41.4	48.0	47.8	47.9
2	How often do you feel as described below? Sad	16.9	4.9	10.8	46.3	35.3	40.6	29.7	48.6	39.4	7.1	11.2	9.2
3	How often do you feel as described below? Cheerful	5.1	6.4	5.8	14.7	22.4	18.7	40.4	45.5	43.1	39.7	25.7	32.5
4	How often do you feel as described below? Happy	2.9	1.5	2.2	13.0	12.4	12.7	42.1	44.3	43.3	41.9	41.8	41.8
5	How often do you feel as described below? Lively	3.3	4.0	3.6	12.8	20.9	16.9	42.4	49.0	45.8	41.5	26.1	33.6
6	How often do you feel as described below? Miserable	60.6	51.5	55.9	24.9	28.1	26.5	10.0	14.8	12.5	4.5	5.6	5.0
7	How often do you feel as described below? Proud	10.9	13.5	12.3	30.1	31.4	30.8	40.8	39.4	40.1	18.1	15.7	16.8
8	How often do you feel as described below? Afraid	20.6	15.2	47.8	47.6	46.4	47.0	24.9	31.2	28.1	6.9	7.2	7.0
**No**	**Item**	**Not at all true of me**			**Slightly true of me**			**Very true of me**			**Extremely true of me**		
		**Boys**	**Girls**	**Total**	**Boys**	**Girls**	**Total**	**Boys**	**Girls**	**Total**	**Boys**	**Girls**	**Total**
**Perception of competition at school**													
1	Students seem to value competition.	12.8	10.6	11.6	35.1	48.4	42.0	40.8	34.6	37.6	11.3	6.4	8.8
2	It seems that students are competing with each other.	11.2	12.5	11.9	36.1	47.2	41.8	42.5	33.8	38.0	10.3	6.5	8.3
3	Students seem to share the feeling that competing with each other is important.	13.0	17.0	15.1	36.7	46.1	41.5	41.5	31.0	36.1	8.9	5.8	7.3
4	Students feel that they are being compared with others.	11.4	12.2	11.8	32.2	37.9	35.2	41.8	36.9	39.3	14.6	13.0	13.8
**Perception of cooperation at school**													
1	Students seem to value cooperation.	11.9	8.8	10.3	28.2	35.8	32.1	47.1	45.0	46.0	12.8	10.5	11.6
2	It seems that students are cooperating with each other.	7.7	6.8	7.2	28.6	32.6	30.6	50.9	50.1	50.5	12.7	10.5	11.6
3	Students seem to share the feeling that cooperating with each other is important.	8.7	7.6	8.1	27.4	32.9	30.2	51.5	49.0	50.2	12.4	10.6	11.5
4	Students feel that they are encouraged to cooperate with others.	10.7	9.5	10.1	27.3	32.4	29.9	46.7	47.4	47.0	15.3	10.8	13.0
**No**	**Item**	**Every lesson**			**Most lessons**			**Some lessons**			**Never of hardly ever**		
		**Boys**	**Girls**	**Total**	**Boys**	**Girls**	**Total**	**Boys**	**Girls**	**Total**	**Boys**	**Girls**	**Total**
**Disciplinary climate**													
1	Students don't listen to what the teacher says.	9.4	7.4	8.4	12.4	15.4	13.9	43.1	44.3	43.7	35.1	32.8	33.9
2	There is noise and disorder.	7.2	5.3	6.2	10.3	11.0	10.7	39.4	39.8	39.6	43.1	44.0	43.5
3	The teacher waits long for students to quiet down.	6.5	5.4	5.9	10.2	10.7	10.4	33.2	35.3	34.3	50.1	48.5	49.3
4	Students cannot work well.	6.4	4.2	5.3	10.8	11.3	11.0	37.8	41.4	39.6	45.0	43.2	44.1
5	Students don't start working for a long time after the lesson begins.	6.6	3.6	5.1	7.3	8.04	7.7	31.0	31.5	31.3	55.0	56.8	55.9
**No**	**Item**	**Not at all true of me**			**Slightly true of me**			**Moderately true of me**			**Very true of me**		
		**Boys**	**Girls**	**Total**	**Boys**	**Girls**	**Total**	**Boys**	**Girls**	**Total**	**Boys**	**Girls**	**Total**
**Goal orientation**													
1	My goal is to learn as much as possible.	6.7	4.01	5.3	24.5	26.2	25.4	26.8	25.9	26.3	28.2	28.8	28.5
2	My goal is to completely master the material presented in my classes.	7.7	6.2	6.9	24.3	27.1	25.7	27.7	26.2	26.9	28.0	27.6	27.8
3	My goal is to understand the content of my classes as thoroughly as possible.	7.4	5.9	6.6	19.8	23.4	21.7	26.6	25.4	25.9	31.2	31.4	31.3
**No**	**Item**		**Min**	**Mean**		**Median**		**Max**	**sd**		**Skew**	**Kurtosis**	
**Life satisfaction**													
1	Overall, how satisfied are you with your life as a whole these days?		0	7.26		8		10	2.72		−0.95	0.1	
**Academic performance**													
1	Science		231.6	482.25		481.87		711.53	77.35		0.01	−0.31	
2	Reading		207.76	484.52		486.29		745.75	88.27		−0.11	−0.27	
3	Mathematics		227.88	491.54		492.26		746.72	76.49		−0.06	−0.21	

*Source: Calculations of the authors based on PISA-2018 in Russia*.

#### Learning Outcomes and Their Relation to Bullying

To estimate the effect of learning outcomes on the probability of becoming a victim of bullying, we built a logistic regression model with an equation presented below.

$$\begin{array}{l} log\frac{P\left(Bullying\right)}{1-P\left(Bullying\right)}=\alpha +\beta_{1}Mathematics+\beta_{2}Reading\\ \;+\beta_{3}Science+\varepsilon \end{array}$$

The results of the model are presented in Table 4. To make the interpretation more meaningful and intuitive, we converted the predictors from the interval to ordinal scale with three levels: low performers, medium performers, and high performers in three core subject domains monitored by PISA: reading, mathematics, and science. PISA defines low performers as schoolchildren that “score below Level 2 on the PISA mathematics, reading, and/or science scales,” as this level is considered the baseline “of proficiency that is required to participate fully in society” (OECD, 2016, p. 37). Schoolchildren who score at Level 1 “can answer questions involving clear directions and requiring a single source of information and simple connections; but they cannot engage in more complex reasoning to solve the kinds of problems that are routinely faced by adults of today in modern societies” (OECD, 2016). The low performers cannot interpret or recognize situations in contexts that require somewhat more than direct inference, being thus unable to “extract relevant information from a single source and make use of a single representational mode” (OECD, 2016, p. 40). Oppositely, high performers showed outstanding results reaching either Level 5 or 6, whereas medium performers are those within levels 2, 3, and 4.

**Table 4:** Regression models.

**Item**	**β**	**Standard error**
**Dependent variable: victim of bullying: yes**		
**Learning outcomes and their relation to bullying**		
Science performance: Low	0.002	0.045
Science performance: Medium	−0.010	0.037
Mathematics performance: Low	−0.027	0.034
Mathematics performance: Medium	−0.009	0.024
Reading performance: Low	0.314^***^	0.035
Reading performance: Medium	0.059^*^	0.028
Constant	0.087	0.0309
Adjusted R-sq.	0.087	
*n*	4,231	
**Psychological climate at school and bullying**		
High level of school belonging	0.055^***^	0.015
Positive disciplinary climate	−0.121^***^	0.011
School environment: Cooperative	−0.085^***^	0.011
School environment: Competitive	0.103^***^	0.01
Constant	0.276	0.010
Adjusted R-sq.	0.047	
*n*	6,298	
**Dependent variable: Emotional states and psychosocial traits**		
**Emotion: Joyful**		
Victim of bullying: Yes	−0.105^***^	0.015
Constant	0.500	0.006
Adjusted R-sq.	0.007	
*n*	6,471	
**Emotion: Happy**		
Victim of bullying: Yes	−0.092^***^	0.015
Constant	0.439	0.007
Adjusted R-sq.	0.005	
*n*	6,506	
**Emotion: Cheerful**		
Victim of bullying: Yes	−0.013	0.014
Constant	0.328	0.007
Adjusted R-sq.	0.000	
*n*	6,485	
**Emotion: Miserable**		
Victim of bullying: Yes	0.084^***^	0.0066
Constant	0.034	0.003
Adjusted R-sq.	0.024	
*n*	6,482	
**Emotion: Afraid**		
Victim of bullying: Yes	0.073^***^	0.008
Constant	0.057	0.004
Adjusted R-sq.	0.013	
*n*	6,457	
**Emotion: Sad**		
Victim of bullying: Yes	0.088^***^	0.009
Constant	0.077	0.004
Adjusted R-sq.	0.015	
*n*	6,457	
**Emotion: Lively**		
Victim of bullying: Yes	−0.020	0.014
Constant	0.340	0.006
Adjusted R-sq.	0.000	
*n*	6,481	
**Emotion: Proud**		
Victim of bullying: Yes	0.035^**^	0.011
Constant	0.162	0.005
Adjusted R-sq.	0.001	
*n*	6,480	
**Life satisfaction: Low**		
Victim of bullying: Yes	0.088^***^	0.014
Constant	0.288	0.006
Adjusted R-sq.	0.006	
*n*	6,476	
**Eudaemonia: High**		
Victim of bullying: Yes	−0.079^***^	0.015
Constant	0.624	0.007
Adjusted R-sq.	0.004	
*n*	6,533	
**Competitivness: Low**		
Victim of bullying: Yes	0.051^***^	0.014
Constant	0.319	0.007
Adjusted R-sq.	0.0019	
*n*	6,536	
**Fear of failure: High**		
Victim of bullying: Yes	0.113^***^	0.014
Constant	0.314	0.007
Adjusted R-sq.	0.009	
*n*	6,507	
**Goal orientation: High**		
Victim of bullying: Yes	−0.062^***^	0.014
Constant	0.311	0.007
Adjusted R-sq.	0.003	
*n*	5,951	
**Task mastery: High**		
Victim of bullying: Yes	−0.097428^***^	0.015
Constant	0.537	0.007
Adjusted R-sq.	0.006	
*n*	6,519	
**Attitude toward bullying: High**		
Victim of bullying: Yes	−0.095^***^	0.013
Constant	0.273	0.006
Adjusted R-sq.	0.008	
*n*	6,478	

* *p < 0.1*,

** *p < 0.05*,

*** *p < 0.01*.

*Source: Calculations of the authors based on PISA-2018 in Russia*.

**Figure 3:**
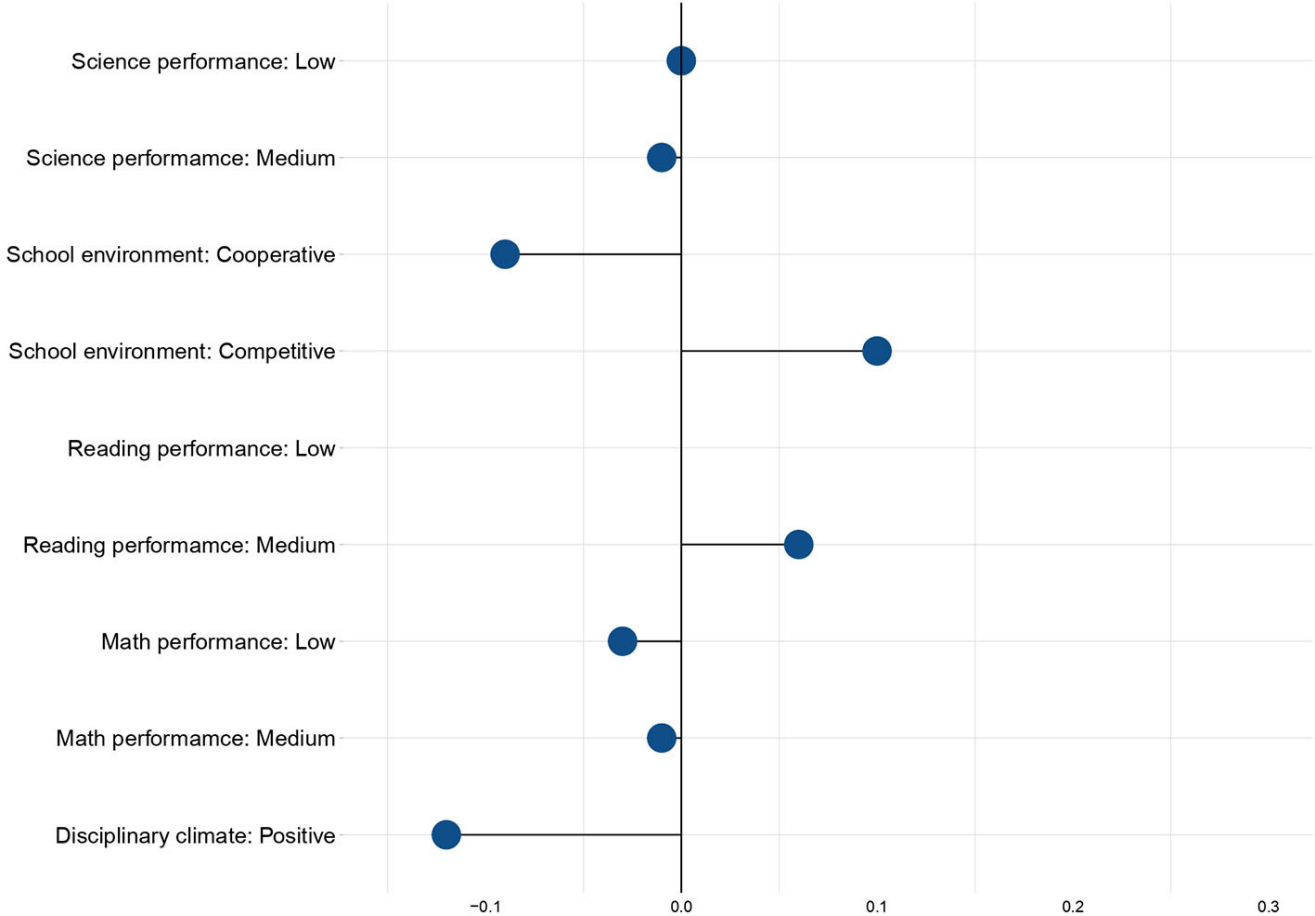
Marginal effects of learning outcomes and the psychological environment of the school on the occurrence of bullying victimization. Source: Calculations of the authors based on PISA-2018 in Russia.

The logits calculated for all three groups across the three domains and presented in Table 4 were converted into probabilities and plotted as marginal effects in Figure 3. High performers were taken as a reference group, and therefore, all marginal effects are presented in relation to the schoolchildren on Levels 5 and 6 in each cognitive test. The results suggest that statistically significant effects of reading performance predict the probability of becoming a victim of bullying. Medium achievers in the reading performance are 5% less likely to become bullying victims than low achievers. The probability is even higher for the group of high achievers, accounting for 27%.

#### Psychological Climate at School and Bullying

PISA provides some variables that could serve as useful proxies for the psychological environment in schools. These variables include disciplinary climate, cooperativeness, and competitiveness of the school environment, and the feeling of children of belonging to school. Using these predictors, we built the following logistic regression model:

$$\begin{array}{l} log\frac{P\left(Bullying\right)}{1-P\left(Bullying\right)}=\alpha +\beta_{1}Disciplinary\;Climate+\\ \;+\beta_{2}Cooperativeness+\\ \;+\beta_{3}Competitiveness+\beta_{4}School\;Belonging+\varepsilon \end{array}$$

Summary results of the model are presented at Table 4, whereas Figure 3 shows the values of marginal effects predicted by the model. All the effects turned out to be highly statistically significant (*p* < 0.01). A competitive school environment demonstrates the highest magnitude of the effect, increasing the probability of bullying by 11%. On the other hand, the likelihood of bullying in schools with a cooperative school environment is 6% lower. It is also clear that a positive disciplinary climate in schools decreases the probability of bullying by 9%. Finally, students who do not demonstrate a high degree of belonging are also 6% more likely to become bullying victims.

#### Emotional States and Psychosocial Traits of the Bullying Victims

**Figure 4:**
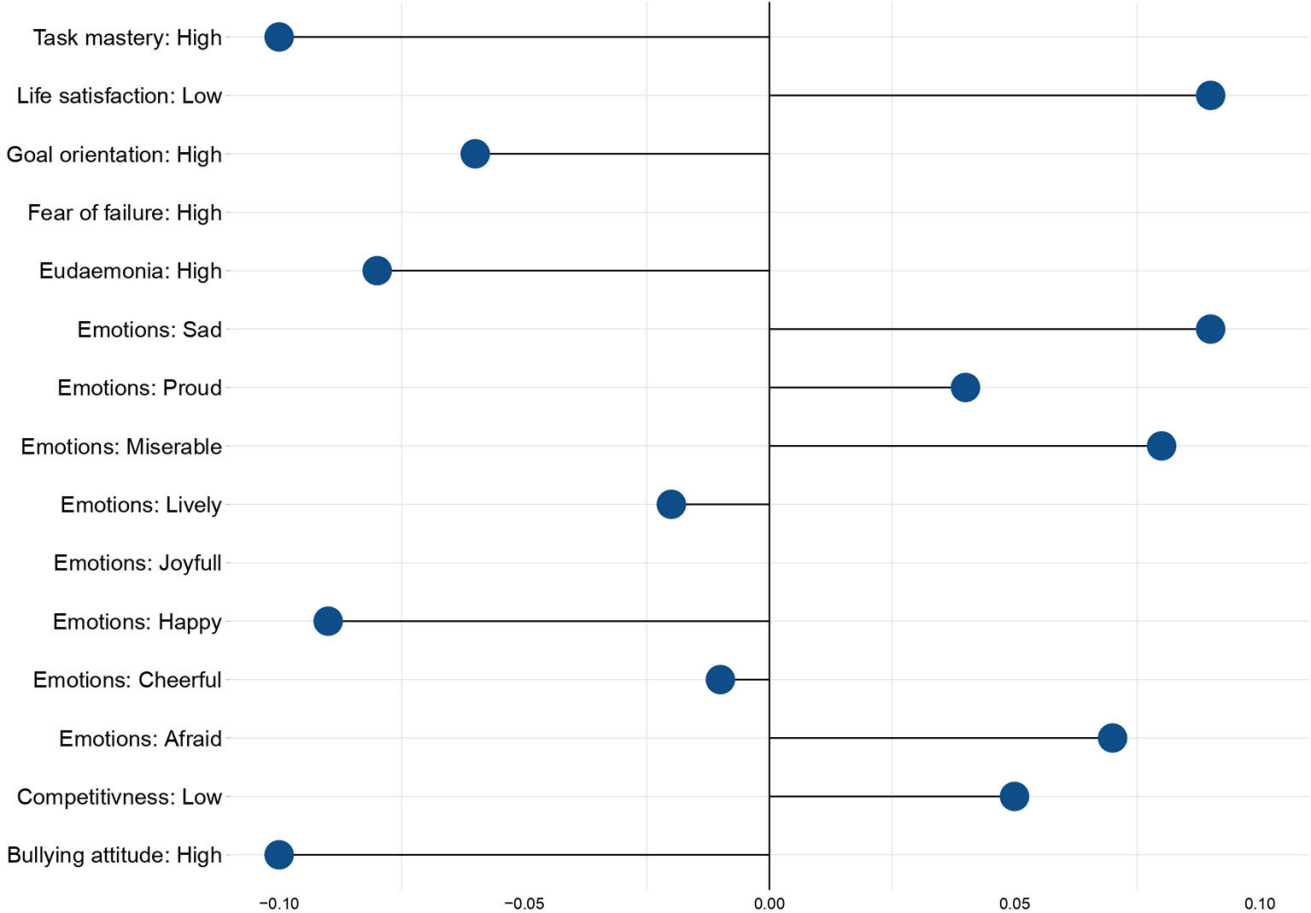
Marginal effects of bullying on the emotional states and psychosocial traits of the victims. Source: Calculations of the authors based on PISA-2018 in Russia.

This part of the analysis looks at victims of bullying, thus aiming to reveal emotional states and psychosocial traits that are most typical for them. With this regard, bullying instead of being a response, became an independent variable of the logistic regression, and the model aimed to estimate the probability of a specific emotional state or psychosocial trait to be typical for bullying victims. We thus ended up running 15 models where bullying predicted the likelihood of a specific emotional states or psychosocial traits. The model thus obtained the following equation:

$$\begin{array}{l} log\frac{P\left(Y\right)}{1-P\left(Y\right)}=\alpha +\beta_{1}Bullying+\;\varepsilon , \end{array}$$

where *P*(*Y*) referred to a probability of a schoolchild to have a certain psychosocial trait or experience very frequently one of eight emotions reported in PISA, accounting for an effect of bullying. The results of these regressions are presented in Figure 4.

As was mentioned earlier, PISA asks students to assess how frequently they feel joyful, happy, cheerful, miserable, afraid, sad, lively, and proud. Logistic regression modeling identified statistically significant effects (*p* < 0.001) of bullying on the occurrence of almost all of the outlined, except cheerfulness and liveliness. The most potent positive effect of bullying is observed in connection with fearfulness, misery, and sadness. Victims of bullying have a higher probability of experiencing these emotions than other students (with marginal effects equal to 7, 8, and 9%, respectively). Bullying is also negatively associated with joy and happiness, which means that bullying victims are 11% less likely to report joy and 9% less likely to report happiness.

Not surprisingly, bullying also shapes both attitudes and behavioral patterns of its victims. As such, the marginal effect of having low life satisfaction levels equals 10% amongst bullying victims. Conversely, bullying victims are less likely to have high eudaemonia levels, a condition defined by PISA as a sense of meaning in life. Also, victims of bullying are 11% more likely to experience a high fear of failure. They are 5% more likely to be found among least competitive schoolchildren, which shows their low ambitions in reaching goals and objectives; The marginal effect of high task mastery equals −10%, which means that bullied schoolchildren are less likely to reach the objectives set.

Finally, the most surprising conclusion refers to the attitude of bullying victims toward bullying itself. As such, victims of bullying are 10% less likely to be among those schoolchildren who have an explicitly negative attitude to bullying.

## Discussion

### Limitations of the Study

The study has some limitations imposed by the data. It appears essential to understand how bullying changes over time and how it transitions from primary to secondary school. However, since PISA collects data from schoolchildren in the last grade of lower secondary school, it does not provide an age variation that would be enough to make this kind of inference. Furthermore, we cannot discount that schoolchildren often become victims of bullying due to their appearance, which involves excess weight, functional difficulties, or even disabilities (Sweeting and West, 2001; Hill, 2017; Pinquart, 2017; Su, 2021). Unfortunately, PISA does not collect anthropometric data from children. Finally, due to the lack of data, it appears impossible to examine the influence of family environment as well as relationships amongst family members on bullying.

### Discussion

The results of our analysis suggest that one out of six 15-year-old children in Russian secondary schools is a victim of bullying. This result is substantially higher than one received in a measurement carried out within “Health Behavior in School-aged Children (HBSC)” study in 2014, which was supported by the World Health Organization (WHO, 2014). The measurement results suggest that up to 13% of schoolchildren aged 15 years are bullying victims in Russia (WHO, 2014). However, the difference in numbers is explained by the fact that the WHO-supported survey looked at a wider age group, and the prevalence of bullying in a younger age is lower than in adolescence.

In many ways, our findings go in line with the data from the last PISA report (OECD, 2019a). As such, the pattern that boys and low-achieving students of both sexes tend to report bullying more often than girls and high-achieving students of both sexes, holds for OECD countries, too. “On an average across OECD countries, students who reported being bullied at least a few times a month scored 21 points lower in reading than students who did not report so, after accounting for socio-economic status” (OECD, 2019a, p. 46). Furthermore, calculations on Russian data also go in line with the OECD countries as bullied students tend to report feeling sad, scared and less satisfied with life, and demonstrate a weaker sense of school belonging than their peers who are less bullied.

The earlier studies also confirmed the prevalence of verbal bullying over other type (Glazyrina et al., 2017). Proceedings from the study carried out in 2011, state that verbal bullying is typically expressed as offensive words, rumors, unreasonable blame, threats, or personal insults, which emphasizes the fact that almost one-third of all cases of verbal bullying ever reported comes from teachers. According to our results, verbal bullying is followed by the relational type, whereas the measurement made by Glazyrina et al. (2017) suggests that the prevalence of physical bullying is second after verbal. It leads to the conclusion that since 2011 there has been a marked shift to psychological, indirect forms of bullying.

In this perspective, our findings go in line with the results of another study with a comprehensive geographical coverage in Russia. This research reveals that social aggression expressed in inappropriate gestures and offensive comments dominates physical aggression (Rean and Novikova, 2019). Since it appears challenging to monitor and sanction psychological violence in opposition to the physical type, which is also very easy to prove, the former becomes more attractive for perpetrators. The lack of any legal framework to regulate psychological violence and its subjective, personal character contribute to the spread of verbal bullying and its prevalence over physical aggression.

One of the bullying aspects that are uniquely specific for the Russian context refers to the reporting of bullying, highlighting significant differences in the perception of bullying by students and teachers. Existing evidence suggests that students agree that bullying should not be reported (Khanolainen et al., 2020). This in turn means that the problem of bullying tends to be severely underestimated by teachers and parents. It results in a significant difference in the perception of bullying by students and teachers, whereas “the majority of teachers indicated either seeing no bullying or only seeing bullying rarely as a justifiable reaction to provocation,” students reported bullying regularly (Khanolainen et al., 2020, p. 1).

Analysis of the socio-demographic profile of victims enabled us to understand the composition of this group by several key dimensions. From the gender perspective, we revealed that boys are more likely to become bullying victims. It makes sense in this context to appeal to the study of Butovskaya and Rusakova (2016), which adds to our results by stating that victimization of girls peaks when they are about 13 years old and then gradually reduces, whereas victimization of boys remains on the same level approximately till they turn 16. Psychophysiological factors explain these differences well. Girls enter into puberty earlier, with the period being accompanied by the secretion of sexual hormones such as testosterone (Copeland et al., 2019; Fassler et al., 2019). Being unsynchronized in their physiological development, girls pass this phase earlier. Strong dependence of aggression levels amongst adolescents on sexual hormones (Finkelstein et al., 1997; Ramirez, 2003) explains the higher prevalence of boys amongst bullying victims. However, the prevalence of boys over girls is not exceptionally high; therefore, it is not gender *per se* but a combination of different psychosocial factors that predict the propensity to bullying (Bochaver and Khlomov, 2013; Shalaginova et al., 2019).

Schoolchildren from low-status groups also bear a certain risk of becoming bullying victims. As has been mentioned, more than 40% of bullying victims belong to 20% of families with the lowest index of economic, social, and cultural status, which also goes in line with other studies (WHO, 2014; Tippett and Wolke, 2015; Butovskaya and Rusakova, 2016; Rean and Novikova, 2019). The stigma associated with belonging to families with a lowest standing is exacerbated at school, and other classmates use it to highlight their dominance (Rean and Novikova, 2019; Vorontsov, 2020). However, it does not mean that bullying is a function of low-status dispositions. Even children from families with high economic, social, and cultural standing can become bullying victims. However, prevention strategies should refer to the so-called rural poor, i.e., children from the most impoverished families in rural areas. Our findings underline that in about 70% of cases victims of bullying reside in rural areas or small towns with a population under 100,000 inhabitants.

The relation of learning outcomes to bullying points out that low achieving students bear the highest risk of being bullied. When it comes to reading skills, in comparison to the high performers, the probability of a low performing schoolchild becoming a bullying victim is almost by 30% higher. The regression could not identify statistically significant effects of subject-specific performance in mathematics and science, which has a clear explanation. Reading test requires a schoolchild to actualize the psychological processes of meta-cognition critical for any analytical activity and thus goes far beyond classroom needs, assessing “literacy skills needed for individual growth, educational success, economic participation and citizenship” and emphasizing the “ability to locate, access, understand and reflect on all kinds of information” which is essential “to participate fully in our knowledge-based society” (OECD, 2019b, p. 22). In this context, a reading test serves as a good proxy for general intelligence and analytical thinking ability, including such literacy skills as “finding, selecting, interpreting, integrating and evaluating information from the full range of texts associated with situations that extend beyond the classroom” (OECD, 2019b). High achievement in this area presumes skills crucial for cognitive activity and social adaptation. It thus allows high-achieving students in reading to avoid situations when bullying is directed at them.

On the other hand, low performers in reading when not reaching even the baseline level of skills necessary to participate in society fully, also lack skills of social communication and adaptation. With this in mind, insignificant effects of science and mathematics are not surprising: children who cannot go beyond direct inferences cannot be achievers in mathematics or science. The results of PISA in 2015 suggest that low performance is rarely limited to one subject, and there is a high overlap between low achievers in all three cognitive domains (OECD, 2016, p. 40).

The regression analysis of variables of the school psychological environment—disciplinary climate, cooperativeness and competitiveness of the school environment, and schoolchildren' feeling of school belonging reveals that they impact the risk of becoming a bullying victim. Whereas, many scholars have mentioned the importance of the psychosocial factors in bullying prevention, our findings indicate its four specific aspects that should draw the focus of specialists while organizing prevention measures and remedial work.

The study also shows that bullying victims have a higher probability of experiencing such negative emotions as fearfulness, misery, and sadness; on the opposite, they have a lower probability of experiencing such positive emotions as joy and happiness. The bullying victims report fewer positive emotions while compared to people on average.

The study also indicates that adolescent bullying vulnerability affects their traits, for example, reduces the level of eudaimonia. Such adolescents experience fear and failures; they are less competitive and often fail to achieve their objectives. The set of the indicated above features characterizes Russian bullied adolescence as persons with an insufficiently mature personality.

Finally, the research has found that bullying victims tend to abstain from expressing a negative attitude toward bullying and do not feel sorry for the victims, proving the possibility of a victim–bully roles switching or combination. This goes in line with the results of other studies that examined whether prior bullying victimization leads to bullying perpetration in the longitudinal perspective (Camodeca et al., 2002; Jose et al., 2012). It is suggested that the switch from one role to another is particularly specific for students with high self-esteem. Another longitudinal study revealed that “students with higher self-esteem were the most likely to engage in future bullying perpetration in response to bullying victimization, while the students with lower self-esteem were the least likely to engage in future bullying perpetration”; as such, for the bully victims with high self-esteem it serves as a possible way to recover threatened egotism (Choi and Park, 2018).

Consequently, we can state that there are two high-risk groups of adolescents in bullying situations, namely : (1) prone to victim behavior and (2) prone to aggressor behavior. That conclusion is consistent with the view of Vorontsov (2020) that not only outsiders but also schoolchildren with social life and friends, i.e., those who seek to raise or preserve their social status among same-age peers at the expense of psychological or physical domination over others, are involved in bullying situations.

The carried out statistical analysis has thus provided a means of identifying the “primary risk group” of bullying victims in the secondary schools of Russia. It should be stressed that the research presents statistically proven pioneer work as the reading test results of PISA assessment have been first applied to estimate the probability of becoming a bullying victim. Similar research-based data have not been found in a large body of published literature.

## Practical Implications

Our research findings provide valuable information for bullying prevention programs. Programs oriented to creating a comfortable psychological climate at school present clear advantages over those oriented to reducing undesirable social behavior patterns. If antibullying programs aim to ensure the psychological well-being of adolescents, they can be more efficient in dealing with the problems that even go beyond bullying. Instead of focusing on specific negative aspects of school life, they provide ground for an inclusive and psychologically comfortable learning environment that rejects bullying. These areas of work should constitute primary preventive measures.

In the secondary prevention phase, the work should focus on those students who are specifically prone to risks of becoming victims. In other words, it should look at the profile of that 16% of schoolchildren who were identified as bullying victims. With these regards, increasing the learning outcomes by improving the literacy skills of low achieving students should be one of the core areas of work. Low performance in reading that outlines a lack of literacy skills needed to succeed in contemporary society shapes life even beyond schools, and bullying is one of the dimensions where the harmful effects of low achievement become so explicit. Another set of measures should be directed at improving the acceptance of students from low-status groups in the classroom to eliminate the influence of status-related issues on bullying.

Working with the behavior of male students is crucial to develop an appropriate and safe expression of anger, aggression, and other negative emotions as these students are especially prone to physical bullying. It is necessary to teach them to understand the psychological essence of aggression, its characteristics, optimize the interaction of the group, develop cooperation, increase school belonging, self-reflection, increase empathy, and create a healthy emotional space. Antibullying programs should facilitate communication skills crucial for better conflict resolution to mitigate verbal or relational bullying.

Generally, prevention strategies and antibullying programs should emphasize the ways and methods of self-control among adolescents. Creating situations of success, setting an encouraging environment that provides ground for positive emotions, developing awareness, and accepting their feelings are core areas of work. Antibullying programs should also teach socially acceptable ways of expressing aggression, aiming to reduce the verbal, indirect aggression through aggressiveness recognition and its think-aloud protocol, and develop empathy and skills of constructive problem solving and fostering personal maturity.

## Conclusions

Our study suggests that, on an average, one out of six children attending secondary school in Russia becomes a bullying victim. This measure is different from simple descriptive statistics based on the prevalence of different bullying types. To identify amongst schoolchildren who reported bullying those who are victims, we looked at the bullying distribution scores and used k-means clustering to crossvalidate our assumptions. These procedures allowed for concluding that for 16% of all schoolchildren at the Russian secondary school, experienced bullying, with some frequency leading to victimization. The findings of our research also indicate the prevalence of verbal bullying over relational and physical ones.

Decomposition analysis of bullying victims outlines that male schoolchildren experience bullying more often. Although not all bullying victims come from marginalized groups, there are clear status-related considerations. More than 40% of bullying victims belong to families with the lowest economic, social, and cultural standing. Furthermore, most of the bullying victims (70%) reside in villages or sparsely populated towns.

Analysis of factors predicting bullying also presents reasons for concern. We identified the relationship between learning outcomes in reading and bullying victimization, which presents high risks for low achieving schoolchildren. Considering the PISA framework, those who do not possess the necessary literacy skills to succeed in life are also likelier to be socially excluded and victimized.

The psychological environment at school forms another group of factors behind bullying. Victimization is more likely to occur in a competitive school environment and, logically, less likely to occur in the cooperative one. Therefore, schoolchildren without a strong feeling of school belonging are also likelier to be bullied. However, our findings highlight that a positive disciplinary climate mitigates victimization. These conclusions provide ground for prevention efforts, and school psychologists and social pedagogues obtain a specific role in monitoring the psychological environment of the classroom.

Our study suggests that bullying substantially affects the psychological well-being of a schoolchild. Bullying provokes negative emotions like fearfulness, misery, and sadness amongst victims. Furthermore, it causes rarer experiences of positive emotions compared to other schoolchildren. These peculiarities are crucial in elaborating bullying prevention programs that should compensate for the deficit of positive emotions amongst the victims and eliminate the harmful effects of the negative ones. The adverse effects of bullying, however, go beyond the emotional states. The bullying victims tend to have lower eudaemonia levels, outlining that they avoid reflecting the sense of meaning in life. They also are more likely to have a low level of life satisfaction in comparison to other schoolchildren.

Finally, one of the critical findings of this study suggests that bullying victims could become perpetrators in other contexts. The analysis pointed out that bullying victims are less likely to share negative attitudes toward bullying and empathize with other bullying victims. It allows for hypothesizing that one person could potentially switch or combine victim–bully roles, and future research on bullying in Russian schools should focus on this aspect more.

Considering this, primary prevention measures should address issues related to the school environment creating a friendly and pleasant atmosphere. The measures aimed to create a positive learning environment would be more efficient by eliminating the conditions in which bullying occurs instead of dealing with its negative consequences and undesirable behaviors. The secondary phase of antibullying programs should take into account emotional states and psychosocial factors of bullying victims to help them overcome frustration and stigmatization caused by bullying, thus ensuring that they can fully participate in the social life of the school and beyond, without risks of being victimized again.

## Data Availability Statement

Publicly available datasets were analyzed in this study. This data can be found at: https://www.oecd.org/pisa/data/.

## Author Contributions

GA and LD are the major authors who developed the initial manuscript. AB reviewed the literature and together with VK drafted the practical implications and prevention strategies. SK and VE contributed to the data processing and analysis. IA revised the final manuscript. All authors contributed to the article and approved the submitted version.

## Conflict of Interest

The authors declare that the research was conducted in the absence of any commercial or financial relationships that could be construed as a potential conflict of interest.

## Publisher's Note

All claims expressed in this article are solely those of the authors and do not necessarily represent those of their affiliated organizations, or those of the publisher, the editors and the reviewers. Any product that may be evaluated in this article, or claim that may be made by its manufacturer, is not guaranteed or endorsed by the publisher.
